# Editorial: Commonalities and Differences in Vestibular and Auditory Pathways

**DOI:** 10.3389/fnins.2022.876798

**Published:** 2022-03-23

**Authors:** Soroush G. Sadeghi, Gwenaëlle S. G. Géléoc

**Affiliations:** ^1^Center for Hearing and Deafness, Department of Communicative Disorders and Science, University at Buffalo, Buffalo, NY, United States; ^2^Boston Children's Hospital and Harvard Medical School, Boston, MA, United States

**Keywords:** vestibular, auditory, hair cells, afferent innervation, efferent innervation, ototoxicity, planar polarity

At the core of the inner ear sensory organs are hair cells capable of detecting nanometer-scale motion induced by movement of the endolymph, either by sound or head movement. Hair cells convert mechanical input into electro-chemical signals (Corey and Hudspeth, 1979) which are transmitted to and interpreted by the central nervous system. While the auditory and vestibular sensory organs in the inner ear have a common evolutionary origin and share many features, the two systems also have many differences that specializes them for appropriate coding of different sensory modalities. For example, transmembrane channel-like Tmc1 and Tmc2 are expressed in auditory and vestibular hair cells of the inner ear where they form the pore of the mechanotransduction channel (Kawashima et al., 2011; Pan et al., 2013). While Tmc2 is only transiently expressed in the developing cochlea, its expression persists in vestibular hair cells (Kawashima et al., 2011). This Research Topic highlights some of the recent advances in the auditory and vestibular fields, with both original research and review articles. One of the aims was also to provide a comparison between vestibular and auditory systems through these articles. Below is a brief review of the topics addressed by articles in this collection.

## Planar Polarity of Vestibular and Auditory Hair Cells

One of the key properties, common to both cochlea and labyrinth is the organization or the planar polarity of hair cells. Deans reviews our current understanding of the developmental mechanisms underlying the generation of planar polarity in the inner ear and its functional significance for effective stimulation of hair cells by sound or motion. Tarchini discusses the similarities between mechanisms that provide orientation of hair cells in the maculae of the otolith organs and the cochlea during development. Finally, Simon et al. show the functional importance of this organization by measuring the vestibulo-ocular response in a mouse model (Celsr1 KO mice) with disorganized hair cell polarity in all vestibular end organs. They show a decrease in the gain of the vestibulo-ocular reflex (VOR) in response to stimulation of either canals or otoliths.

## Vestibular and Auditory Hair Cells

Typically, depolarization of hair cells results in opening of voltage sensitive calcium channels (Ca_V_1.3) and entrance of calcium into the cell, which then activates calcium-sensitive mechanisms of vesicular release of glutamate from the hair cell onto afferent terminals. There is also a non-quantal method of synaptic transmission present in the vestibular periphery, between type I hair cells and their calyx terminals, which is due to accumulation of potassium ions and glutamate (Contini et al., 2012, 2017, 2020; Songer and Eatock, 2013; Sadeghi et al., 2014). This is in contrast to fast and large multivesicular quantal synaptic transmission between inner hair cells and afferent terminals in the cochlea (Keen and Hudspeth, 2006; Grant et al., 2010, 2011; Rutherford et al., 2012; Huang and Moser, 2018; Niwa et al., 2021). Interestingly, loss of Ca_V_1.3 in a KO mouse model results in deafness, but no clear signs of imbalance. Using patch clamp recording from hair cells in these mice, Manca et al. show that the calcium current is about 20% of the normal value in both cochlear inner hair cells and type I and II vestibular hair cells. This suggests an abnormal vesicular transmission. However, the hair cells show a normal development of potassium channels, resulting in accumulation of potassium between type I hair cells and their calyx afferent terminals with hair cell depolarization. While these mice most likely have compensatory changes in their central vestibular pathways, the results of this study also suggest a role for the non-quantal transmission in the vestibular periphery in the lack of apparent imbalance in these mice.

## Vestibular and Auditory Afferents

Vestibular afferents have specific innervation patterns in the periphery and those with irregular resting discharges innervate the central regions of the cristae and maculae where more type I hair cells and calyx terminals are present (Goldberg et al., 1984, 1990; Baird et al., 1988; Fernandez et al., 1988, 1995; Desai et al., 2005a,b). In the mammalian vestibular periphery, calyx terminals specifically express calretinin as their calcium binding protein (Desmadryl and Dechesne, 1992; Dechesne et al., 1994; Lysakowski et al., 2011). Reichenberger et al. show that in frogs, which as anamniotes lack calyx terminals and type I hair cells, calretinin is expressed only in the ganglion cells that innervate hair cells in the auditory end organ and not in the vestibular end organ. The authors discuss the functional significance of these findings in relation to requirements for encoding different sensory modalities (i.e., movement vs. acoustic stimulation). Kalluri reports that even though auditory and vestibular afferents serve different functions, they are similar in the range of resting potentials, voltage thresholds, current thresholds, input resistances, and first-spike latencies in mice.

## Vestibular and Auditory Efferent Innervation

Efferent inputs are required for normal function of the vestibular (Hubner et al., 2015; Raghu et al., 2019) and auditory pathways (Glowatzki and Fuchs, 2000; Maison et al., 2006; Johnson et al., 2013) and play a role in aging (Lauer et al., 2012; Zachary and Fuchs, 2015; Fuchs and Lauer, 2019; Boero et al., 2020; Vicencio-Jimenez et al., 2021). In this collection, Lorincz et al. provide a detailed map of central and peripheral projections of cholinergic vestibular efferent neurons in mice, using state of the art methods, including transgenic mice and Cre-dependent adeno-associated virus mediated expression of fluorescent reporters. Their results show a rich dendritic arborization toward the vestibular nuclei (suggesting that they receive their inputs mainly from these nuclei) and a dominant contralateral vestibular efferent innervation in this mammalian model. In another article, Lee et al. compare the effectiveness of intratympanic, intracochlear, and systemic application of different cholinergic agonists and antagonists and their effect on the resting discharge of afferents. In another article in this collection, using patch clamp recordings Meredith and Rennie study the role of dopamine, another candidate efferent neurotransmitter in both the auditory and vestibular periphery. They show that activation of D2 dopaminergic receptors decreases the amplitude of Na^+^ currents in calyx terminals, with possible inhibitory effects on afferent firing. This is in contrast to the excitatory effects of activation of cholinergic (Poppi et al., 2020; Ramakrishna et al., 2021; Schneider et al., 2021) and GABA-B (Ramakrishna and Sadeghi, 2020) receptors in the calyx. Finally, a review article by Cullen and Wei provides a comparative summary of our current knowledge about vestibular efferents across different animal models, sensory/motor signals carried by efferents, and their possible function compared to auditory efferents.

## Development and Aging of the Inner Ear

On the topic of developmental changes, Quinn et al. study the expression of Na^+^ channels in the developing hair cells of the human fetal inner ear neuroepithelia. They show that the number of vestibular and auditory hair cells that express these channels decreases with age and while both TTX-sensitive and TTX-insensitive/resistant currents are present in developing hair cells, there is a differential role for TTX-sensitive Nav1.6 (SCN8A) channels in the vestibular neuroepithelium compared to Nav1.7 (SCN9A) in the cochlea. Rabbitt and Holman study another part of the peripheral circuitry, the supporting cells and show a calcium signal that can be modulated by purinergic or cholinergic inputs. This signal can play a role in the development/maturation of the vestibular end organs and pathways, similar to what has been proposed in the cochlea (Glowatzki and Fuchs, 2000).

Two articles address aging in the inner ear. Paplou et al. review and compare what we know about age-related hearing and vestibular loss. This review provides similarities and differences in the prevalence of age-related changes in the two systems, possible common underlying mechanisms (such as inflammation, oxidative stress, and genetic factors), and changes at the cellular level in the peripheral sensory organs of the two systems. Andresen et al. provide further information regarding age-related changes in expression of melanin pigmentation in the vestibular and auditory periphery in mouse and human samples. With increasing age, they find an increase in pigmentation in the stria vascularis, but little change in the vestibular end organ. Whether this change in pigmentation plays any protective role remains to be studied.

## Stimulation by Vibration vs. Sound

The vestibular system has traditionally been tested with slow sinusoidal rotations. More recent clinical tests (e.g., vestibular evoked myogenic potentials or VEMPs) use higher frequency bone vibrations or sound for stimulating the vestibular end organs. Previous studies have shown that afferents, particularly irregular ones that innervate the otoliths or canals can be stimulated by vibration or sound (Zhu et al., 2011, 2014; Curthoys et al., 2014, 2019). In this collection, Curthoys et al. use a mix of data and modeling approaches to provide a broad overview of VEMP tests and their neural pathways, the mechanics of otolith high frequency responses or its “seismometer mode,” and the phase locking of afferents during such fast movements. Finally, they compare these high frequency otolith responses to those of the auditory periphery. Chen et al. explore the response to sound at the level of the vestibular nuclei (VN), abducens, and eye movements in rats. They show that the stimulation results in activity in VN neurons that receive inputs from canals and/or otoliths. Interestingly, while the stimulation results in the activity of less than half of the recorded abducens neurons, it results in eye movements with horizontal and vertical components.

## Cisplatin Ototoxicity

Prayuenyong et al. review the evidence that shows cisplatin, a chemotoxic drug used for treating different cancers differentially affects the cochlea and has little effect on the labyrinth. This is evidenced by loss of hair cells in the cochlea (particularly the outer hair cells), but almost no effect on vestibular hair cells. Consistent with this pattern of peripheral damage, while a proportion of patients complain about hearing loss and tinnitus there is little evidence for any abnormal vestibular tests. The authors propose different hypotheses for this selective effect, including easier transfer through the stria vascularis into the cochlear endolymph, higher endocochlear potential that might help drive the drug into auditory hair cells, and higher concentrations of the drug in the cochlear perilymph.

## Conclusion

Taken together, the collection of studies in this Research Topic addresses the similarities and differences between the auditory and vestibular systems at different levels. Review articles provide a summary of the current state and new research articles add to this knowledge. Hopefully, this Research Topic will motivate researchers to consider the similarities of two systems, as it brings about the potential to uncover basic mechanisms that may be conserved in evolution. On the other hand, the articles also emphasize the differences between the two systems, which shows the mechanisms developed to optimize them for their specific functions. Some of the articles also highlight the interaction between basic science findings and their clinical applications, holding promise for identifying novel testing paradigms or therapeutic approaches in the inner ear, with more targeted effects and fewer side effects.

## Author Contributions

Both authors contributed to this editorial and approved the submitted version.

## Funding

SS received funding from Capita Foundation.

## Conflict of Interest

The authors declare that the research was conducted in the absence of any commercial or financial relationships that could be construed as a potential conflict of interest.

## Publisher's Note

All claims expressed in this article are solely those of the authors and do not necessarily represent those of their affiliated organizations, or those of the publisher, the editors and the reviewers. Any product that may be evaluated in this article, or claim that may be made by its manufacturer, is not guaranteed or endorsed by the publisher.
